# Variable Branching Pattern of Tibial Nerve in the Tarsal Tunnel: A Gross Anatomical Study With Clinical Implications

**DOI:** 10.7759/cureus.13729

**Published:** 2021-03-06

**Authors:** Suranjana Banik, Leon R Guria

**Affiliations:** 1 Anatomy, All India Institute of Medical Sciences, Bhubaneswar, IND; 2 Anatomy, Regional Institute of Medical Sciences, Imphal, IND

**Keywords:** tarsal tunnel syndrome, tibial nerve, nerve block, nerve graft, nerve compression, high tibial osteotomy, electromyography, paralysis, flexor retinaculum, medial malleolar calcaneal axis

## Abstract

Introduction

Tibial nerve is a larger component of the sciatic nerve. It arises from ventral branches (Anterior Division) - L4, L5, S1-S3. Then it travels along the distal border of the popliteus muscle, deep to gastrocnemius and soleus. In the leg, it is accompanied by the posterior tibial vessels and lies in the tarsal tunnel. It divides into the medial calcaneal nerve at the ankle, medial, and lateral plantar nerves under the flexor retinaculum. It carries sensory information. It can adapt to repeated forces and undergo stretch especially in ankle joint dorsiflexion and inversion of the foot. Compression of the tibial nerve in the tarsal tunnel can cause tarsal tunnel syndrome. Many surgical procedures need tibial nerve block which demands detailed knowledge of its variation.

Materials and methods

The study was cross-sectional and included lower limbs of five embalmed cadavers and 10 separate cadaveric lower limbs and was performed in the Department of Anatomy of Regional Institute of Medical Sciences, Imphal, India. The reference line (1 cm width) joining two landmarks medial malleolus and medial tubercle of calcaneus called the mideo-malleolar-calcaneal axis was determined and bifurcation of the tibial nerve was classified with respect to the axis.

Results

The tibial nerve in all the cases also crossed the posterior tibial vessels. In 11 cases (55%), the bifurcation of the tibial nerve was proximal to the mideo-malleolar-calcaneal axis with a mean distance of 1.86 cm above the axis, and thus comprising the maximum Type I category. Type II category, having bifurcation at the level of the axis, was found in six (30%) cases. Type III category, having three (15%) cases, was recorded to have bifurcation at a mean distance of 1.16 cm.

Conclusion

Proper anatomical knowledge of tibial nerve branching is required to prevent surgical complications, effective nerve block, procurement of tibial nerve graft.

## Introduction

The tibial nerve, which is the larger component of the sciatic nerve, is derived from the ventral branches (anterior divisions) of the fourth and fifth lumbar and first to third sacral ventral rami. It descends along the back of the thigh and the popliteal fossa to the distal border of the popliteus muscle. It further passes deep to gastrocnemius and soleus muscles, and then anterior to the arch of soleus muscle with the popliteal artery. In the leg, the tibial nerve travels with posterior tibial vessels and lies in a fibro-osseous tunnel called the tarsal tunnel. Medially the tunnel is bounded by the flexor retinaculum, laterally by the posterior aspect of talus and calcaneum, and anteriorly by the medial malleolus. The tibial nerve divides into medial and lateral plantar nerves under the flexor retinaculum [1].

Medial calcaneal nerve arises at the ankle, pierces the flexor retinaculum, and supplies the posterior and lower surface of the heel [2] whereas the medial and lateral plantar branches innervate the medial plantar and the lateral plantar areas of the foot, respectively, and carry sensory information from those areas [3].

The tibial nerve can undergo stretch during the movement or different positions of the lower limb, especially, ankle joint dorsiflexion and inversion of the foot. As a result, the nerve has to adapt itself to such changes through its mechanical properties. It can adapt to repeated forces and can slide in relation to the surrounding tissues [4,5].

Compression of the tibial nerve in the tarsal tunnel due to its narrowing can cause tarsal tunnel syndrome [6]. Moreover many surgical procedures, as well as a tibial nerve block in this area, requires detailed knowledge of the variations in the level of division of the nerve.

The present study was done to analyze the topographic anatomy of the tibial nerve and its respective branches in the ankle in relation to the tarsal tunnel and its applied clinical significance.

## Materials and methods

The study was performed in the Department of Anatomy of Regional Institute of Medical Sciences on lower limbs of five embalmed cadavers and 10 separate cadaveric lower limbs that are used for undergraduate and postgraduate teaching purposes. Due to resource constraint only 20 limbs were available during the study period of one year and consecutive sampling technique was adopted. The limbs were dissected and the skin, superficial fascia was removed 30 cm proximal to the medial malleolus and it was continued distally to the plantar surface in each foot. Dissection was started from the popliteal fossa, continued downwards at the back of the leg until the flexor retinaculum. Each foot was placed in the anatomical position and it included the positioning of the foot 90° to the tibia to standardize the measurements.

A reference line (1 cm width) was determined from the two landmarks that are the tip of the medial malleolus (MM) to the medial tubercle of the calcaneus (MTC), because of their prominence and easy palpation on physical examination. This medio-malleolar-calcaneal (MMC) axis also represents the inferior edge of the flexor retinaculum and as a result, the tarsal tunnel [7]. The tarsal tunnel was defined by extending 2 cm proximal and distal to the axis [8]. The bifurcation of the tibial nerve was classified with respect to the defined axis. Type I represents that the bifurcation is proximal to this axis. Type II represents that the bifurcation is at the axis and type III represents that the bifurcation is distal to the axis [7].

Distances were measured using a digital Vernier caliper with 0.001 mm accuracy. Detailed recordings of the typical findings were recorded, and photographs of more significant anatomic dissection were captured. Mean values and standard deviation of the measurements of the right and left sides for each foot were statistically calculated. For statistical analysis, SPSS (Statistical Package for the Social Sciences) 21 version (IBM Corp., Armonk, NY) and Microsoft Excel (Microsoft® Corp., Redmond, WA) were used.

## Results

**Figure 1:**
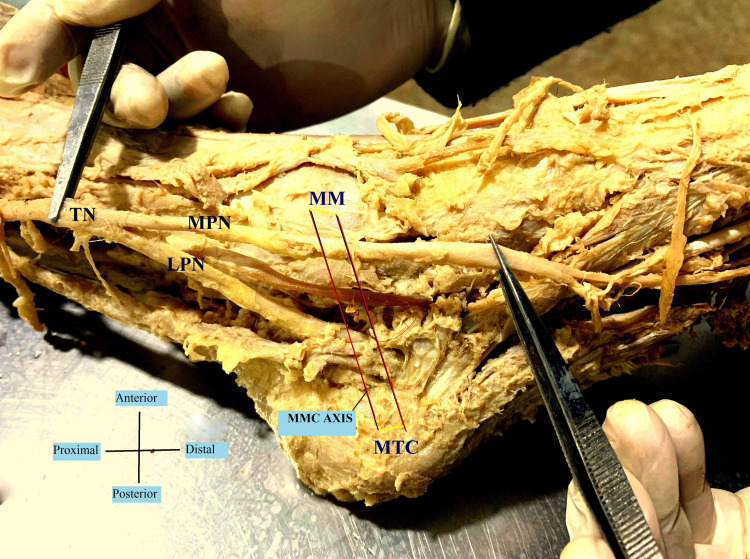
Bifurcation of the tibial nerve proximal to MMC (Medio-Malleolar-Calcaneal) axis – Type I TN- Tibial Nerve; MPN- Medial Plantar Nerve; LPN- Lateral Plantar Nerve; MM- Medial Malleolus; MTC- Medial Tubercle of Calcaneus.

**Figure 2:**
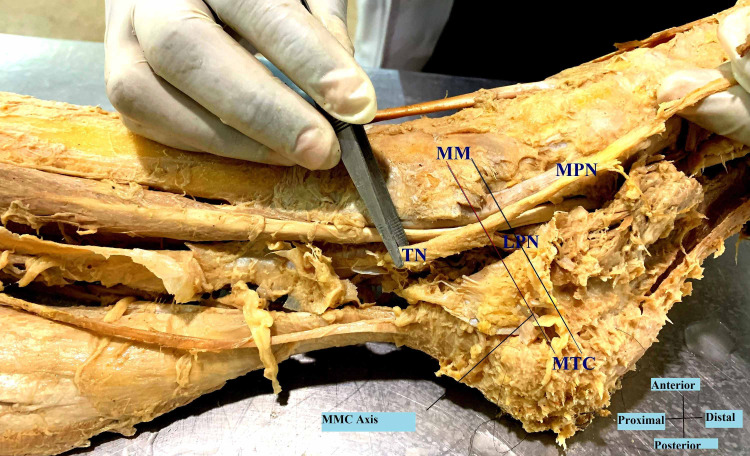
Bifurcation of the tibial nerve at the MMC (Medio-Malleolar-Calcaneal) axis – Type II TN- Tibial Nerve; MPN- Medial Plantar Nerve; LPN- Lateral Plantar Nerve; MM- Medial Malleolus; MTC- Medial Tubercle of Calcaneus.

**Figure 3:**
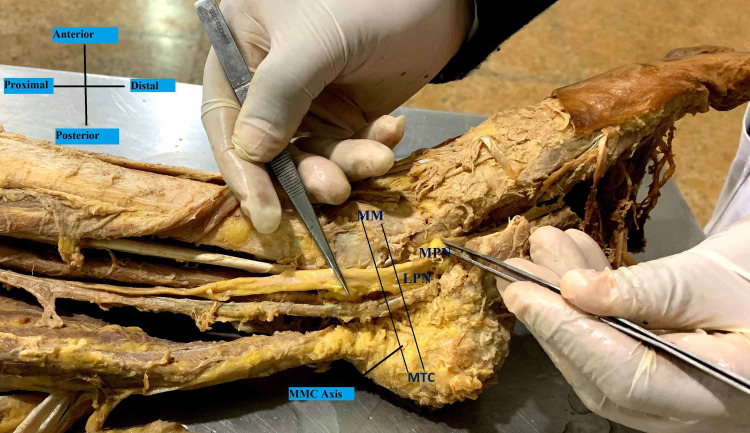
Bifurcation of the tibial nerve distal to the MMC (Medio-Malleolar-Calcaneal) axis – Type III MPN- Medial Plantar Nerve; LPN- Lateral Plantar Nerve; MM- Medial Malleolus; MTC- Medial Tubercle of Calcaneus

In the examined 20 lower limb specimens of this study, the tibial nerve divided into the usual median and lateral plantar nerves in a tarsal tunnel in all (100%). The tibial nerve in all the cases also crossed the posterior tibial vessels. In 11 cases (55%), the bifurcation of the tibial nerve was proximal to the medio-malleolar-calcaneal axis with a mean distance of 1.86 cm above the axis (Tables 1, 2), and thus comprising the maximum Type I category (Figure 1). Type II category (Figure 2) having bifurcation at the level of the axis was found in six (30%) cases. Type III category, having three (15%) cases, was recorded to have bifurcation at a mean distance of 1.16 cm distal to the axis (Figure 3). The arrangement of vessels was normal and no accessory innervations causing anomalous branching pattern was noticed. All the muscles had usual innervations.

**Table 1:** Distribution of categories of bifurcation with reference to medio-malleolar-calcaneal axis MMC: Medio-Malleolar-Calcaneal

Types	Number of Cases (n) = 20	Percentage (%)	Mean of Distance from MMC axis (cm)	Standard deviation
Type I	11	55	1.86	1.46
Type II	6	30	At the axis	-------
Type III	3	15	1.16	0.28

**Table 2:** Distance of bifurcation of tibial nerve from the medio-malleolar-calcaneal axis

Cadaveric Lower Limb	Right Lower Limb	Left Lower Limb
Cadaver 1	Type I, 1 cm proximal to axis	Type I, 1 cm proximal to axis
Cadaver 2	Type II, at axis	Type I, 6 cm proximal to axis
Cadaver 3	Type I, 2 cm proximal to axis	Type II, at axis
Cadaver 4	Type I, 2 cm proximal to axis	Type III, 1 cm distal to axis
Cadaver 5	Type II, at axis	Type I, 1.5 cm proximal to axis
Individual Limbs	Right Lower Limb	Left Lower Limb
Limb 1		Type III, 1.5 cm distal to axis
Limb 2	Type II, at the axis	
Limb 3		Type I, 1 cm proximal to axis
Limb 4		Type I, 2.5 cm proximal to axis
Limb 5	Type I, 1 cm proximal to axis	
Limb 6		Type I, 1 cm proximal to axis
Limb 7	Type II, at the axis	
Limb 8		Type II, at the axis
Limb 9	Type I, 1.5 cm proximal to axis	
Limb 10		Type III, 1 cm distal to axis

## Discussion

The knowledge of the anatomical variation of tibial nerve bifurcation is important for the diagnosis and treatment of clinical conditions like tarsal tunnel syndrome or for surgical procedures like external nailing of tarsal bones. In a study by Andreasen Struijk et al., they found that functional electrical stimulation systems for correction of foot drop and providing standing support requires designing and implanting nerve cuff electrodes that can measure the sensory information from all the three terminal branches of the tibial nerve. They thus estimated the branching pattern, the fascicular separability, and the fascicular size of the tibial nerve posterior to the medial malleolar calcaneal axis and found high dispersion in branching pattern but mostly all (80%) within the tarsal tunnel [3].

As per Louisia and Masquelet [9], and Dellon and Mackinnon [10], the bifurcation occurred in the tarsal tunnel in 73% and 95% of the cases, respectively. Ninety-three percent and 90% of the bifurcations were in the tarsal tunnel as found by Havel et al. [11] and Davis and Schon [12]. In our study, 100% branching occurred within the tarsal tunnel.

Moreover, the branches of medial and lateral plantar nerve as well as medial calcaneal nerve, nerve to abductor digiti minimi, and motor branches to abductor hallucis and to joints, vessels and skin take origin directly from the tibial nerve. Surgeons need a thorough knowledge of the variations to avoid inadvertent cutting of nerve branches, which might be dangerous [13].

The anomalous medial plantar nerve may also lead to compression in the region of the heel causing unnecessary pain in the heel along with edema and inflammation of synovial sheaths leading to entrapment of the tibial nerve [14].

Davis and Schon have shown in their study that there is a discrepancy between the results obtained from clinical tests and electromyography studies due to anomalous branching pattern of tibial nerve above flexor retinaculum [12].

In the present study, we found that the tibial nerve bifurcated approximately 1.86 cm above the mideo-malleolar-calcaneal axis. Now as per Burton et al. tibial nerve block was reported as a safe and effective method for controlling pain after outpatient surgery of hallux valgus [15]. A tibial nerve block is also important for postoperative analgesia after total knee arthroplasty in combination with the femoral nerve block. This block also provides good analgesia in children after knee and ankle surgery [16]. So 100% success rate of nerve block can be guaranteed only when the higher branching knowledge, as well as average value, will be available. Knowledge of the bifurcation distance can be effectively used for planning minimal skin incisions in the case of tibial nerve decompression by the release of known anatomical compression points, the soleus arch, and the tarsal tunnel [17].

Kurtoglu et al. stated that a high division of the tibial nerve was associated with an accessory flexor digitorum muscle [18]. We did not come across such findings but probably a larger sample size can help in better evaluation of this. The tibial nerve is often iatrogenically injured during fibular graft harvest, high tibial osteotomy, and fascial release procedures [4]. The topographical knowledge will also help to procure the allogenic vascularised tibial nerve graft after limb salvage along with nerve bank storages for future use [19].

This is the first study of this kind in the North-East Indian population. The major limitation of the study is the small sample size due to resource constraint. The study can be replicated in different ethnic group and with a larger sample size to estimate statistical significant difference in variations.

## Conclusions

The tibial nerve is involved in many clinical conditions starting from poliomyelitis, nerve compression syndromes, tuberculosis, leprosy, idiopathic heterotopic ossification. Damage to either of the branches can cause paralysis of the three muscles supplied by it. So anatomical knowledge of the motor branching will help to reduce surgical complications involving this region. It will also help to reduce the discrepancies in clinical and electromyographical correlation studies. Effective nerve blocks will be hastened. Nerve grafts can be used effectively for nervous tissue banks and limb salvage surgeries can be hassle-free and successful.
